# Superstrength of nanograined steel with nanoscale intermetallic precipitates transformed from shock-compressed martensitic steel

**DOI:** 10.1038/srep36810

**Published:** 2016-11-28

**Authors:** Hailiang Yu, Ming Yan, Cheng Lu, Anh Kiet Tieu, Huijun Li, Qiang Zhu, Ajit Godbole, Jintao Li, Lihong Su, Charlie Kong

**Affiliations:** 1 School of Mechanical, Materials & Mechatronics Engineering, University of Wollongong, NSW 2500, Australia; 2 Department of Materials Science and Engineering, South University of Science and Technology of China, Shenzhen 518055, China; 3 Electron Microscope Unit, University of New South Wales, Sydney, NSW 2052, Australia

## Abstract

An increasing number of industrial applications need superstrength steels. It is known that refined grains and nanoscale precipitates can increase strength. The hardest martensitic steel reported to date is C0.8 steel, whose nanohardness can reach 11.9 GPa through incremental interstitial solid solution strengthening. Here we report a nanograined (NG) steel dispersed with nanoscale precipitates which has an extraordinarily high hardness of 19.1 GPa. The NG steel (shock-compressed Armox 500T steel) was obtained under these conditions: high strain rate of 1.2 μs^−1^, high temperature rise rate of 600 Kμs^−1^ and high pressure of 17 GPa. The mean grain size achieved was 39 nm and reinforcing precipitates were indexed in the NG steel. The strength of the NG steel is expected to be ~3950 MPa. The discovery of the NG steel offers a general pathway for designing new advanced steel materials with exceptional hardness and excellent strength.

Ultrafine-grained/nanograined(UFG/NG) metallic materials have superior mechanical properties. Such materials have therefore attracted increasing attentions from materials scientists and structural engineers. There have been many reports on nonferrous NG materials based on aluminium^1^,^2^,^3^, copper^4^,^5^,^6^,^7^ and nickel^8^,^9^, fabricated using severe plastic deformation (SPD) techniques^10^. However, to date, there have been only a limited number of studies on NG steels^11^,^12^,^13^, although steel is the most widely used metal in industrial applications.

Among the various types of strengthening mechanisms and methods, grain refinement is regarded as the most appropriate method to simultaneously improve strength and toughness. UFG/NG steels exhibit outstanding mechanical properties including high strength-to-weight ratio and great resistance to wear. In body-centered cubic (*bcc*) ferrous alloys, thermomechanical controlled processing in conjunction with microalloying can refine the grain size down to less than 5 μm^13^. Recently, by using a combination of plastic deformation and short-duration annealing, the grain size of steel was refined down to ~250 nm^11^.

In this study, we report a significantly refined grain size and an extraordinarily high hardness achieved by ballistic test to induce shock-compression in Armox 500T armor steel (see methods). Grain refinement and precipitation-strengthening are the two major strengthening mechanisms that induce the observed ultrahigh hardness. The study may offer a viable approach to develop ultrahard steels.

## Results

**Figure 1:**
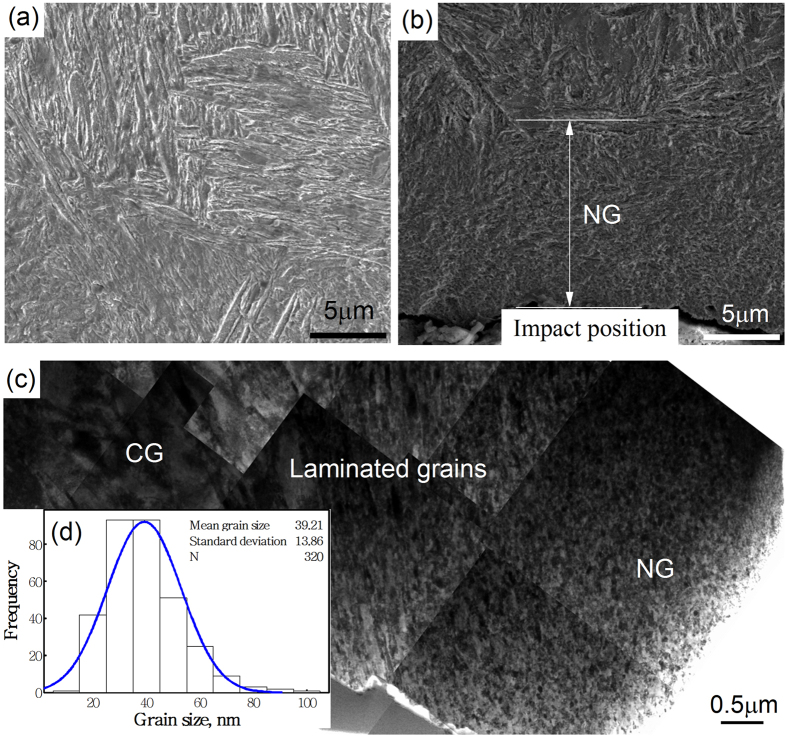
SEM images of (**a**) metal matrix and (**b**) close to the impact position; (**c**) TEM image close to the impact position; (**d**) grain size distribution of nanograins (i.e. in the NG zone).

Figure 1 shows the microstructures of the steel before and after the ballistic test. Figure 1a shows the original microstructure of the tempered martensitic steel. After ballistic test, the microstructure close to the impact position changes into an NG state. The thickness of the NG layer is about 12~15 μm, as indicated in Fig. 1b. Figure 1c shows a TEM image of the region close to the impact position. A NG zone, a laminated grains zone and a coarse-grained (CG) zone can be observed from the impact position (outermost surface) to the metal matrix. Extended Data Fig. 1a–c presents more details of the entire surface area characterized by TEM bright field (BF) images. At the outermost surface of the sample, the original coarse grains have been transformed into equiaxed nanograins (Extended Data Fig. 1c), whose average grain size (measured from 320 grains) is 39 nm, as shown in Fig. 1d. The microstructure of the region between the equiaxed nanograins and CG martensite appears as a laminated structure, as shown in Extended Data Fig. 1b.

**Figure 2:**
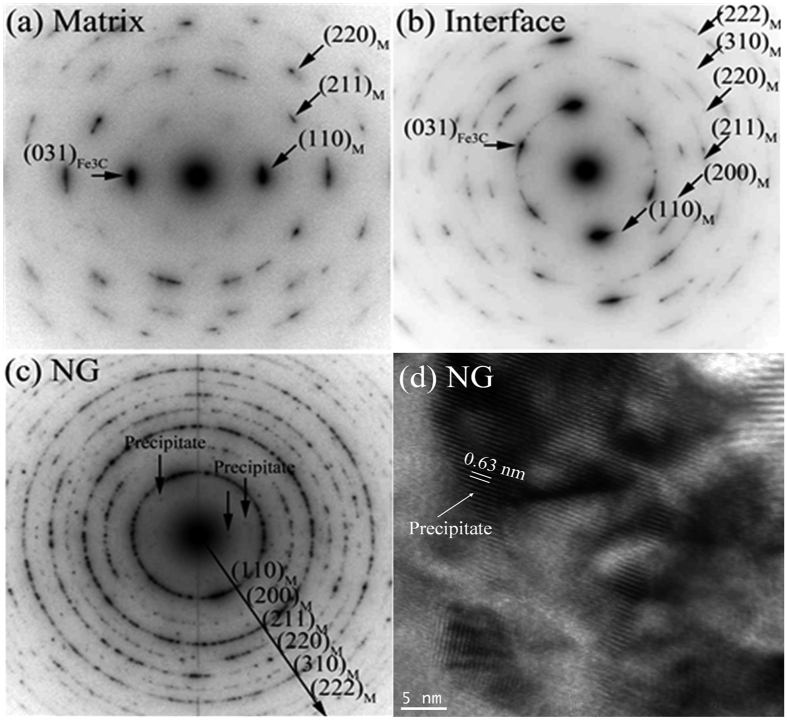
SAED patterns of (**a**) the CG zone, (**b**) the laminated grains zone, and (**c**) the NG zone of the steel after ballistic test, and (**d**) high resolution TEM image of a precipitate newly formed in the NG zone.

**Figure 3:**
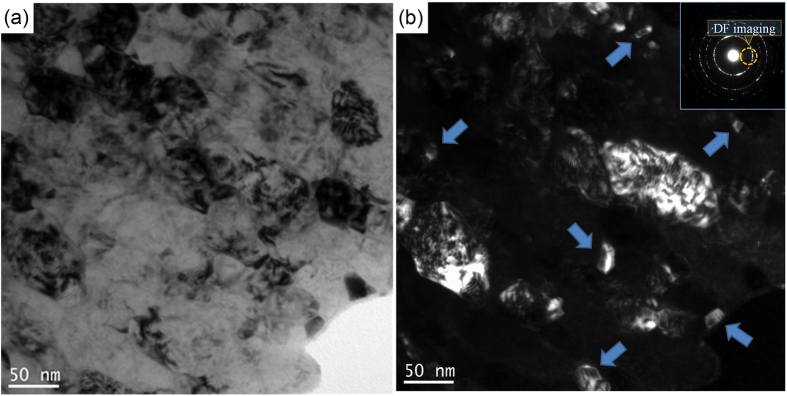
TEM images of precipitates in NG zone. (**a**) bright field (BF) image, (**b**) dark field (DF) image and the corresponding electron diffraction condition. The precipitates are highlighted by arrows.

Further details of selective area electron diffraction (SAED) patterns of the different zones are presented in Fig. 2a–c. The CG zone which corresponds to the metal matrix is mainly composed of the tempered martensitic phase. It is highly possible that iron carbide phase (Fe_3_C) exists in the microstructure. This is evidenced by the extra diffraction spots recorded, which is accompanying the diffraction from the martensitic phase (see Fig. 2a,b and Extended Data Fig. 2). In the SAED image of the NG zone (Fig. 2c and Extended Data Fig. 1c), there can be seen two dramatic differences compared to the original metal matrix:

1. There is great refinement of grain size as indicated by the much more continuous electron diffraction rings compared to those of the CG zone, due to larger number of finer grains involved in the electron diffraction. This is consistent with the results from the TEM-BF imaging (see Extended Data Fig. 1a–c).
2. New phase(s) emerge in the NG zone due to the ballistic processing. This is evidenced by the fact that extra diffraction spots are detected, although their intensities are relatively weak (Fig. 2c). TEM observations (Fig. 3) suggest that there are nanoprecipitates embedded in the matrix. Their size is around 20 nm. High resolution TEM-BF imaging shows that the nanoprecipitates have significantly larger lattice parameters than the steel matrix (Fig. 2d). The interplanar spacing of the precipitate is 0.63 nm while the interplanar spacing of martensitic steel matrix is about 0.28 nm^14^,^15^. The chemical composition and the crystal structure of the nanoprecipitates will be studied in future. In the meantime, electron diffraction from the Fe_3_C phase appears to have fully disappeared, since the related diffraction spots are no longer detected.

**Figure 4:**
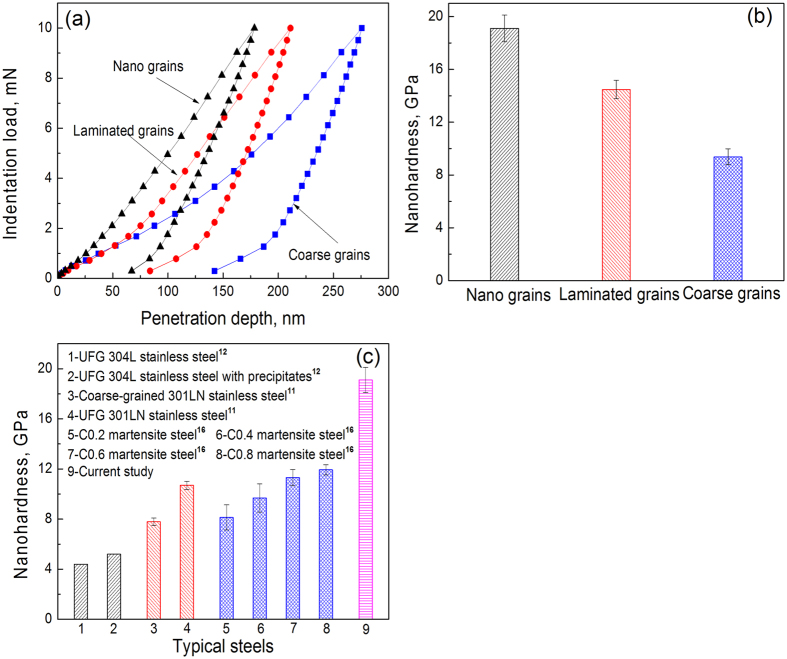
(**a**) load-penetration curves and (**b**) nanohardness of NG zone, laminated grains zone and CG zone, (**c**) comparison of nanohardness of steels in references and current study.

Figure 4a,b show the nanoindentation load-displacement curves and nanohardness at the NG zone, the laminated grains zone and CG zone. Extended Data Fig. 3 shows the related atomic-force microscopy results. In the NG zone, the nanohardness reaches 19.1 GPa. The nanohardness of the original CG layer is around 9.3 GPa, which is characteristic of martensitic steels (8.1 GP for 0.2C martensitic steel, and 9.7 GPa for 0.4C martensitic steel)^16^. The nanohardness of the NG steel nearly doubled the CG value by an increment of ~10 GPa. The yield stress of the original Armox 500 T steel is measured at 1250 MPa. Judging from the relationship between nanohardness (*H*_*n*_) and yield stress (σ_*y*_) (Δσ_*y*_ ≈ 270 Δ*H*_*n*_)^17^, the yield strength of the NG steel might reach 3950 MPa. Figure 4c shows the nanohardness for typical steels and it can be seen that the value in our study is much higher than that of other kinds of steels, including UFG steels. The nanohardness of the ultrafine-grained/nanograined (UFG/NG) 301LN stainless steel can reach 10.7 GPa compared to 7.8 GPa of its coarse-grained (CG) counterpart^11^, and the nanohardness of the UFG 304L stainless steel can be enhanced from 4.4 GPa to 5.2 GPa by precipitation hardening^12^. Thus, the most likely reasons for the greatly enhanced hardness and yield strength are the grain refinement and the precipitation strengthening resulting from the newly formed precipitates.

## Discussion

**Figure 5:**
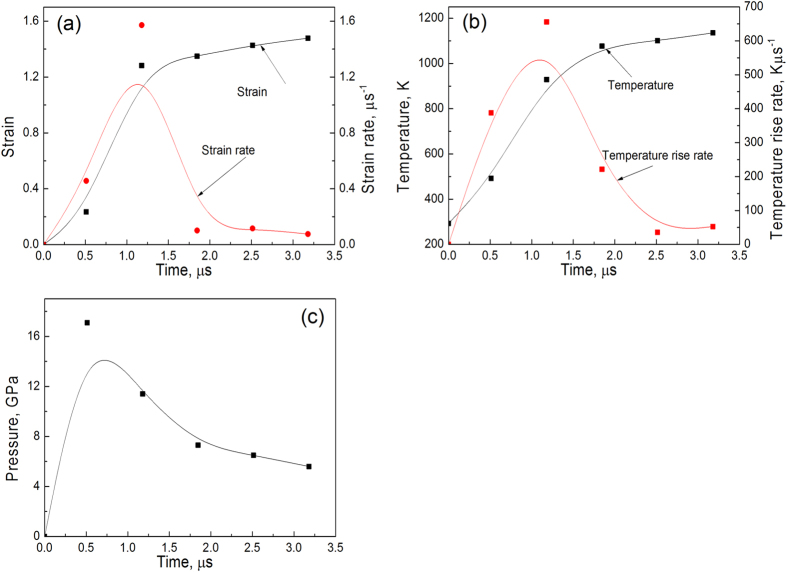
(**a**) strain and strain rate vs time; (**b**) temperature and temperature rise rate vs time; (**c**) pressure vs time. The results were simulated using the finite element method.

As shown in Fig. 1, the CG martensitic steel was transformed into NG steel. In SPD, high equivalent strain and high strain rate can both contribute to grain refinement. The equivalent strain and the strain rate of the ballistic test have been simulated by finite element method and the results are shown in Fig. 5a. It can be seen that the equivalent strain is ~1.2 and is relatively small for SPD processes. It is slightly more than the equivalent strain of one pass equal channel angular pressing (ECAP) processing. Hao *et al*.^18^ reported that the average grain size of a (ferritic+martensitic) steel is about 300–400 nm after four passes of ECAP processing and is much larger than the grain size achieved in this study. There are extensive molecular dynamics simulation results showing that high strain rate deformation could induce grain refinement^19^,^20^. The deformation at higher strain rates will produce more uniform dislocation distribution for the same strain, hinder the formation of discrete dislocation cells, decrease the cell size, and increase misorientation with more dislocations trapped with cell interiors^21^. In the present study, the strain rate reaches a very high value of 1.2 μs^−1^. Thus, we propose that the impact of high strain rate on the grain refinement is much greater compared to that induced by the application of an equivalent strain.

Apart from the strain rate, the deformation temperature also determines the final grain size of materials subjected to SPD^22^,^23^. In our study, a temperature rise has been predicted as shown in Fig. 5b. The temperature of the sample may increase to ~1200 K within a very short period of time, with a temperature rise rate of ~600 Kμs^−1^. It is known that the temperature for the austenite transformation, A_c3_, of the ARMOX 500T steel is ~1059 K^24^, i.e. below 1200 K. This means that the original martensitic phase and iron carbide phase in the microstructure (see Fig. 1a) can fully transform into austenite phase, and then subsequently undergo rapid cooling, during which their grain sizes are further refined from the μm scale to the nm scale. Misra *et al*.^11^ found that annealing 62% cold-rolled Cr-Ni steel at 1173 K for 10 s results in phase reversion from martensite to austenite. In this process, recrystallization of the austenite grains follows coalescence of the sub-grains to form UFG/NG structure with grain size of 250 nm. It is thus proposed that the short annealing at 1200 K also plays a key role in the grain refinement in the current study. Using the hardness model (*H* = *H*_*0*_ + *K*_*H*_*d*^*1/2*^) derived from the Hall-Petch relationship^25^, we have calculated the constant *K*_*H*_ based on the measured grain size data (~28 nm for the NG zone and ~10.0 μm for the CG zone), which gives *K*_*H*_ = 2.0. This is quite close to the value of ~2.4 for 316L steel. It also implies that grain refinement is likely the dominating factor that has significantly enhanced the hardness of the steel after the ballistic processing. In addition, for the metals with surface NGs, there are some reports showed that nano-gradient materials have both high strength and high ductility^26^,^27^.

Apart from the strengthening effect due to the grain refinement, nano precipitates^28^ also contribute to the improvement in the material hardness and strength. There are extensive reports on strain-induced precipitation in steels^29^,^30^. Llanos *et al*.^29^ found that strain-induced precipitation can occur at temperature lower than 1273 K for Nb steel. At the simulated temperature rise for the present study, strain-induced precipitates are highly likely to be formed. Shen *et al*.^31^ found that in shock-compressed SiO_2_, silica transforms into a form of glass which subsequently exhibits ultrafast crystallization within nanoseconds. Mediana^32^ also reported that the nucleation time of precipitates is only 0.05 s when the strain and driving force for phase transformation are high. In the present study, nanoscale precipitates can nucleate and grow during the early cooling stage. The driving force may come from the high momentum passed on through the impact of the bullet. Initially, the highly deformed crystal structure may provide more non-equilibrium nucleation sites to form intermetallic compounds. Once the nucleate size exceeds a critical size, they are stabilized. In addition, we propose that the high pressure of 17 GPa during the ballistic test (Fig. 5c) have also contributed to the formation of these nanoscale precipitates. Figures 2c and 3 show that reinforcing nanoscale particles are formed. These nanoscale precipitates will in turn contribute to the improvement of strength of materials from dispersing strengthening mechanisms such as pinning the grain boundaries and Orowan looping^33^.

Finally, we propose that the high pressure of 17 GPa during the ballistic test (Fig. 5c) may result in phase transition of steel. Takahashi and Bassett^34^ reported that there is a phase transition from α-Fe to ε-Fe when the pressure exceeds ~11 GPa. The ε-Fe phase also has a lattice distance of 1.84 Å, which is close to one of the *d* values we measured (i.e. 1.83 Å). This suggests that under the high pressure, the ε-Fe phase may form as well as one type of nanoprecipitate. Also, there are some reports^35^,^36^ showing that with additional ε-Fe in the matrix, the mechanical properties of materials were improved greatly. Li *et al*.^35^ reported that a TRIP-DP-HEA steel can obtain both high strength and high ductility. For this steel, the partial martensitic transformation of the face-centred cubic (FCC) to hexagonal close-packed phase (HCP, epsilon-Fe) took place upon cooling from the high-temperature single-phase region. This change enables development of a dual-phase (FCC&HCP) microstructure in which both phases obtain the maximum benefit of the solid-solution strengthening effect.

## Methods

A 10 mm thick Armox 500T steel sample was used in this study. This steel is one of the best protection plate steels used in military vehicles, buildings, etc. The chemical composition (wt.%) is: C-0.32, Si-0.4, Mn-1.2, P-0.015, S-0.010, Cr-1.01, Ni-1.81, Mo-0.7, B-0.005 and Fe-balance. A GBC MMA X-ray diffractometer (XRD) with Cu Kα radiation (λ = 1.5418 Å) was used to measure the phase diffraction profiles. The yield stress of the steel was measured at 1250 MPa. A ballistic test with a fragment fired at a speed of 650 m/s was used to induce high strain rate deformation in the steel. After ballistic test, it is expected that the cooling rate would be very fast, in the order of thousand degrees per second.

After the ballistic test, the microstructure of sample was investigated using a Philips CM200 Field Emission Gun Transmission Electron Microscope (FEG/TEM) equipped with a Bruker Energy Dispersive X-ray (EDAX) Spectroscopy system, operating at an accelerating voltage of 200 kV. TEM samples were prepared by focused ion beam (FIB) technique on an FEI xT Nova Nanolab 200 Dual-beam FIB/SEM workstation (Extended Data Fig. 4). The FIB/SEM workstation was also used to observe the microstructure of the sample etched by using a 2% nital solution. Digital Micrograph version 3.7.4 was used in TEM result analysis. The TEM-SAED patterns in Fig. 2 are shown in phase reversal to enhance image contrast. The high resolution TEM analysis was carried out on the ultrathin specimen in a wedge shape with overall thickness less than 100 nm and the thin edge on the top about 30 nm thick. Extra precaution was taken to avoid destructive ion beam damage in the FIB. A thick Au coating of 60 nm, *in-situ* Pt deposition coating of 2 μm and slow process with a fine ion beam were the three key measures used to avoid altering the thermal sensitive microstructure with localized beam heating. The bright field images were taken with the smallest objective aperture available on the TEM. The lattice images were taken with the second largest objective aperture to allow multiple diffracted beams to form the interference fringe image of the fine grains at high magnifications. The selected area electron diffraction patterns were also recorded for the regions of interest.

Nanoindentation tests were performed on the cross section of oxide scale using the UMIS nanoindentation system (UMIS2000, CSIRO) with a Berkovich diamond indenter (radius of tip is around 200 nm). The maximum load was chosen as 10 mN in the nanoindentation tests. The square root load control mode was selected where the load was gradually increased until it reached the peak value and then decreased. The indentation load vs displacement curves were continuously recorded by software with a resolution of 75 nN in load and 0.05 nm in depth. The hardness was calculated according to the Oliver and Pharr method using a tip shape correction. The nanohardness (*H*) of samples is calculated by Eq. (1)^37^.


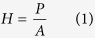


where *A* is the contact area (*A* = 24.5*h*_*p*_^2^). *h*_*p*_ is the penetration depth of the indenter. Before the test the UMIS system was calibrated on a standard fused silicon sample whose elastic modulus and hardness were 72.5 GPa and 9.5 GPa, respectively. After nanoindentation, the residual indents were examined by a Nanoscope IIIA atomic force microscope (AFM, Dimension 3100) in the contact mode with a lateral resolution of 1–5 nm and a vertical resolution of 0.08 nm. The nanoprobe cantilevers were made of silicon nitride (Si_3_N_4_) with a spring constant of 0.06 N/m. Extended Data Fig. 5 shows the nanoindentation matrix. In addition, the SEM images for the nanoindentation matrix were carried out by using JEOL JSM-7001F field emission gun-scanning electron microscope capable of 3 nm spatial resolution operating at 15 kV.

Two-dimensional finite element method was used to calculate the temperature increase and strain rate of the sample during ballistic penetration, using LS-DYNA. In the models, there are 119700 nodes and 211582 elements. The heat capacity of the sheet material was set as 46 J/(gK), and thermal conductivity as 460 W/(mK), the initial temperature of the sheet as 293 K, and the friction coefficient between the bullet and plate was set as 0.1. During deformation, the fraction of mechanical work converted into heat was set at 0.7^38^.

## Additional Information

**How to cite this article**: Yu, H. *et al*. Superstrength of nanograined steel with nanoscale intermetallic precipitates transformed from shock-compressed martensitic steel. *Sci. Rep.* **6**, 36810; doi: 10.1038/srep36810 (2016).

**Publisher's note:** Springer Nature remains neutral with regard to jurisdictional claims in published maps and institutional affiliations.

## Supplementary Material

Supporting Materials
