# Supplementary material for: Superstrength of nanograined steel with nanoscale intermetallic precipitates transformed from shock-compressed martensitic steel
Source: Sci Rep. 2016 Nov 28;6:36810. doi: 10.1038/srep36810 (PMC5125102; doi:10.1038/srep36810)
Supplement: Supporting Materials [file srep36810-s1.doc]

**Superstrength of nanograined steel with nanoscale intermetallic precipitates transformed from shock-compressed martensitic steel**

Hailiang YU^1^, Ming YAN^2^, Cheng LU^1^, Anh Kiet TIEU^1^, Huijun LI^1^, Qiang ZHU^1^, Ajit GODBOLE^1^, Jintao LI^1^, Lihong SU^1^, Charlie KONG^3^


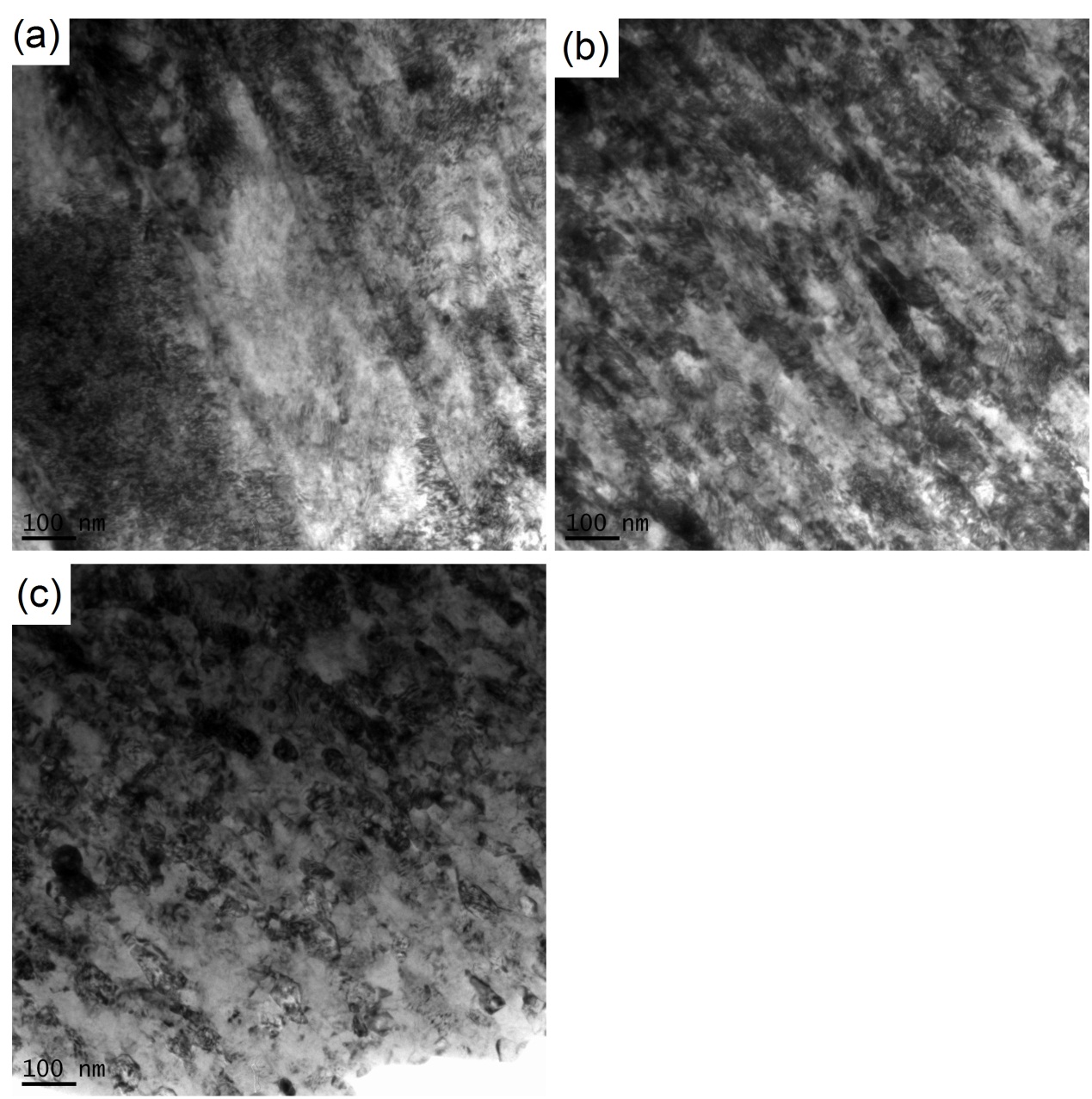


**Extended Data Figure 1 | (a), (b) and (c) for TEM BF images for the coarse grains, laminated grains and nano grains respectively.**


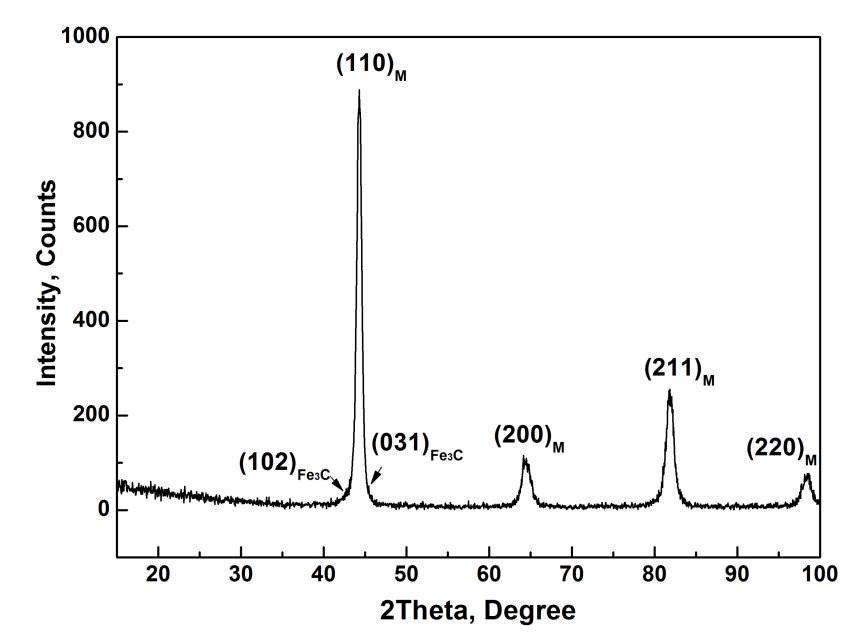


**Extended Data Figure 2 | XRD result of as-received ARMOX 500T steel, showing tempered martensitic structure.**


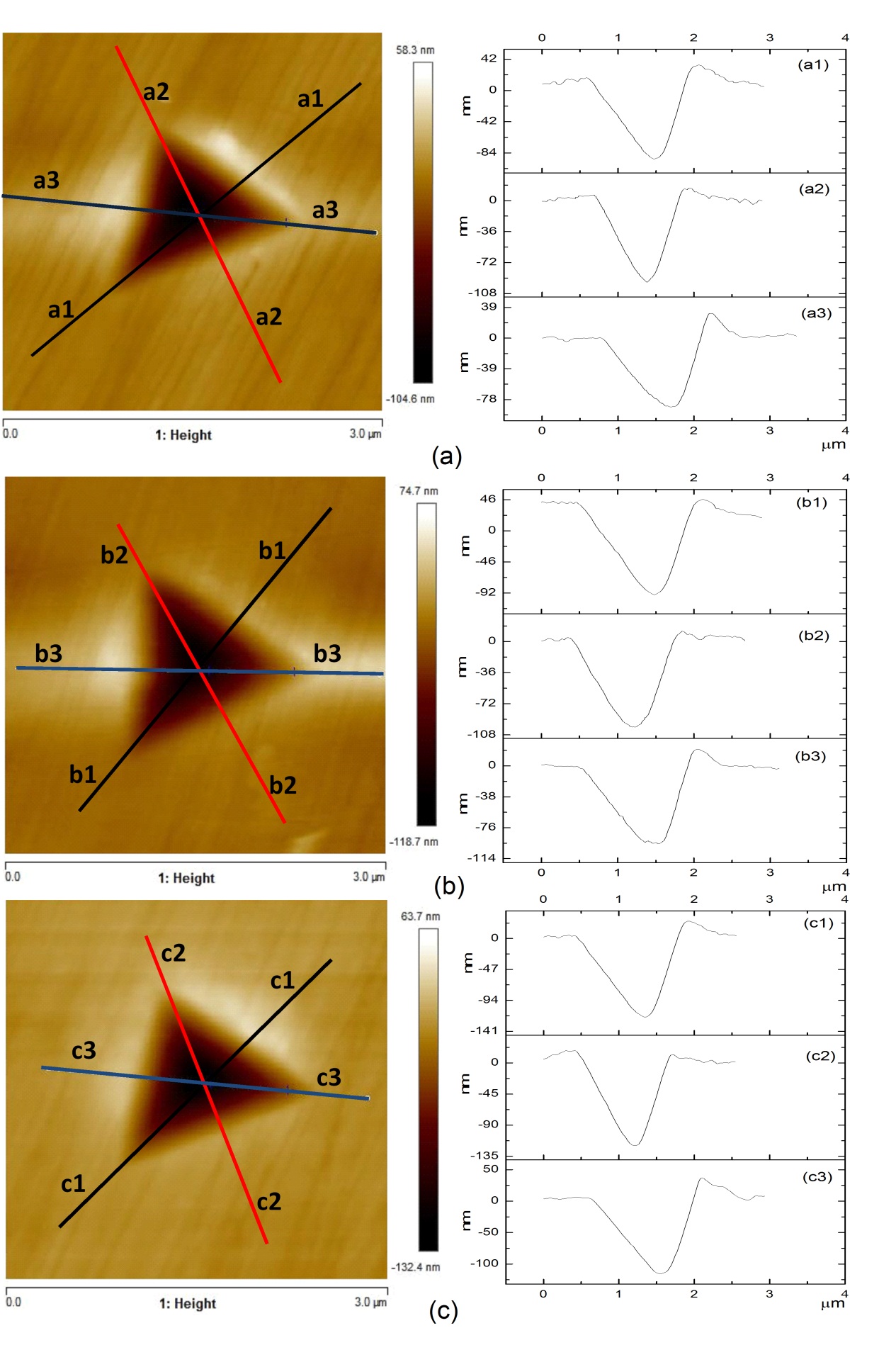


**Extended Data Figure 3 | AFM results for typical nanoindentation tests on the (a) nano grains; (b) laminated grains; (c) coarse grains.**


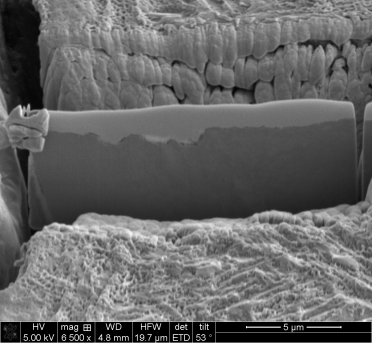


**Extended Data Figure 4 | SEM image of an FIBed sample for TEM analysis.**


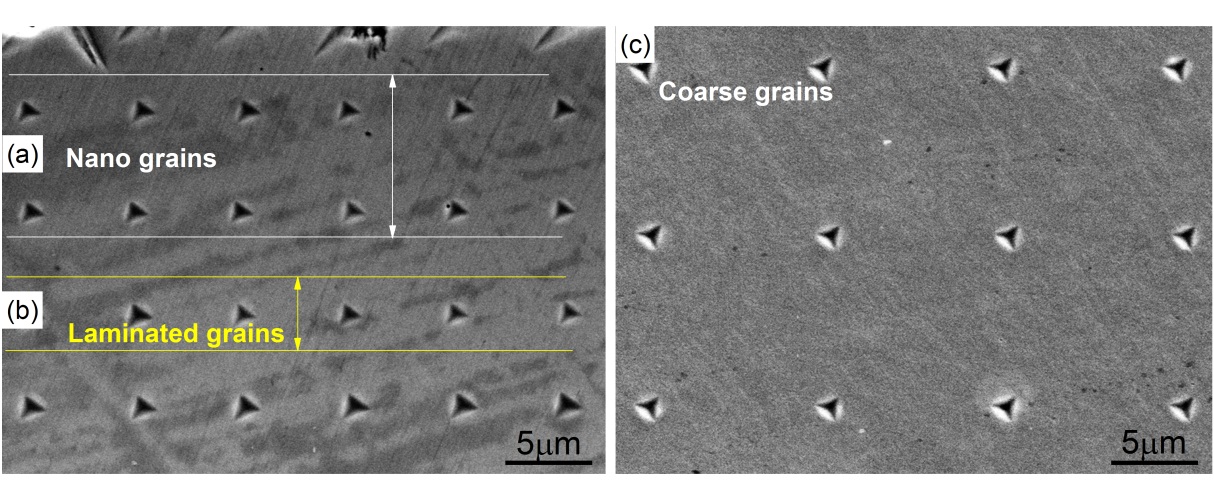


**Extended Data Figure 5 | SEM images for nanoindentation points, showing testing points in the zones of (a) nano grians, (b) laminated grains and (c) coarse grains.**
